# Functionalized Triazine-Based Small Molecules as Efficient Battery Anode Materials

**DOI:** 10.1021/acsomega.5c04787

**Published:** 2025-09-16

**Authors:** Yao-Chih Lu, Febri Baskoro, Meng-Ju Yang, Hung-Ju Yen, Long-Li Lai

**Affiliations:** † Department of Applied Chemistry, 59433 National Chi Nan University, No. 1 University Rd., Puli, Nantou 545, Taiwan; ‡ Material Science and Engineering Research Group, Faculty of Mechanical and Aerospace Engineering, Institut Teknologi Bandung, Jl. Ganesha 10, Bandung 40132, Indonesia; § 38017 Institute of Chemistry, Academia Sinica, 128 Academia Road, Section 2, Nankang, Taipei 11529, Taiwan

## Abstract

Leveraging the remarkable structural versatility and chemical robustness of triazine frameworks, we synthesized two novel triazine-based derivatives—**pOMeAni** and **pOMe2CN**—and explored their potential as high-performance anode materials for lithium-ion batteries (LIBs). To investigate the effect of the active material content, electrodes with two different loadings (20 and 40%) were evaluated. The electrochemical performance showed that the triazine-based anodes exhibited a high specific capacity of up to 459 mA h g^–1^ at 100 mA g^–1^ after 100 cycles with nearly 99% Coulombic efficiency. Interestingly, the triazine-based anodes show excellent rate capability and cycling stability at a high current density of 2000 mA g^–1^ up to 2000 cycles with a peak capacity of 445 mA h g^–1^. The high current and long-term operation of the triazine-based anode outperformed the commercial graphite-based anode, emphasizing its potential as an alternative anode of LIBs. Furthermore, the mechanistic and kinetic studies on the triazine-based anode reveal structure/performance relationships that shine light on the structural design of high-performance anode materials. These results not only highlight the immense lithium storage potential of triazine derivatives but also open new avenues for the design of next-generation organic electrode materials in sustainable energy storage systems.

## Graphical abstract

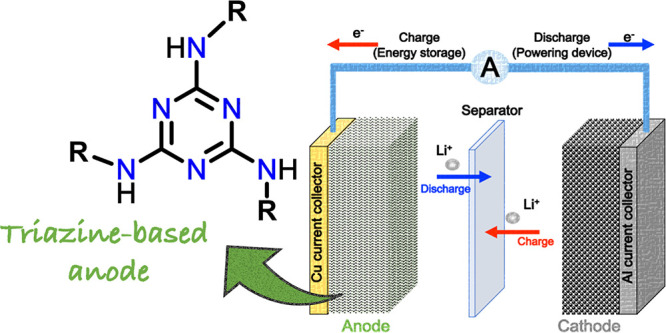

## Introduction

Nowadays, with booming technology and the changes in our daily lives, our needs for effective and high-capacity energy storage devices are ever-growing.^1^ Along with the rise in sustainability awareness,^2^ many related problems such as material sources and environmental concerns have been shown. Therefore, there is an urgent need to find alternative solutions to address these issues. Lithium-ion batteries (LIBs) are a major solution to energy issues due to their light weight, low self-discharge, longer cycling lifespan, and relatively straightforward manufacturing processes.^3–7^ They have diverse applications, including portable devices, electric vehicles (EVs), and power supplement of AI memory devices. These applications are a hot topic these days; specifically, EVs, as a novel industry, are still in the growing stage.^8^

Carbon-based graphite is most widely used as a battery anode. The layered structure in graphite allows lithium-ion intercalation during the charging process, accommodating 1 Li atom per 6 C atoms, corresponding to the formula LiC_6_. This Li–C coordination results in its low theoretical capacity of only 372 mA h g^–1^ and limits the battery performance.^9^ Such limitations in materials, coupled with environmental and resource constraints, have aroused the attention of researchers to alternative solutions. Toward the promising direction, organic materials have shown potential due to their tunable structures, diverse types, and accessibility from abundant natural resources.^10–15^ Additionally, nitrogen-rich organic materials stand out due to the high affinity of nitrogen for lithium ions,^16^ which makes them suitable for providing rich active sites. Nitrogen-containing materials, such as azo-based and covalent triazine frameworks (CTFs),^17^ have been widely explored. Among them, triazine is a promising electrode material due to its advantages. Its derivatives offer high thermal and chemical stability, while its planar aromatic structure enables strong π–π interactions, forming stable frameworks. The triazine structure is also customizable, provides a high density of active sites for energy storage, and is synthesized using easily accessible reactants and solvents, further enhancing its sustainability.^18–21^ These properties improve the energy density, reduce energy gaps, and offer eco-friendly alternatives to traditional inorganic materials, making triazine-based materials ideal for LIB anodes. Several studies have shown the potential of triazine-based materials for LIB anodes. Zhang and co-workers reported a triazine-based conjugated porous polymer capable of delivering specific capacities of 565 and 375 mA h g^–^^1^ at 100 and 1000 mA g^–^^1^, respectively, due to efficient Li-binding in the triazine moiety.^22^ Moreover, porous CTFs have also been successfully synthesized and evaluated as LIB anodes. The study shows that a CTF synthesized from 1,3,5-triazine-2,4,6-triamine and piperazine-1,4-dicarbaldehyde via the solvothermal method (CIN-1) was capable of delivering a specific capacity of 292 mA h g^–1^ up to 250 cycles under 100 mA g^–1^.^23^ The low specific capacity of CIN-1 was due to an increase in resistance in the bulk structure.^23^ Additionally, the triazine microporous polymer ACT delivers a specific discharge capacity of 247 mA h g^–1^ at 5000 mA g^–1^ current density over 4000 cycles, suggesting high structural stability.^24^ While the aforementioned studies highlight the potential of triazine-based materials for LIB anodes, small triazine compounds have received less attention. Herein, we therefore introduce two triazine-based materials functionalized with distinct functional groups: methoxy and nitrile groups. These materials are named **pOMeAni** and **pOMe2CN**, respectively. In this study, both materials are applied as LIB anodes and subjected to a series of electrochemical measurements for comparison.

## Results and Discussion

### Materials Synthesis and Characterization

Two triazine-based materials, **pOMeAni** and **pOMe2CN**, were synthesized according to the method shown in Scheme 1. Both triazine-based materials were centered on a 2,4,6-triamino-1,3,5-triazine backbone with phenyl groups as linkers and were functionalized by two different substituents, methoxy and nitrile groups, at their *para*-positions. NMR, MALDI-TOF mass, and Fourier transform infrared (FT-IR) spectroscopy techniques were employed to derive structural information on these two compounds (Figures 1a and S1–S5). As shown in Figure 1a, two vibrational peak at 3350–3500 cm^–1^ can be attributed to N–H stretching, while two peaks at 3350–3500 cm^–1^ result from C–H stretching in aromatic and phenyl groups. Furthermore, the strong peaks at around 1230–1030 cm^–1^ were attributed to the C–O vibration of the methoxy groups. Meanwhile, the C–N aromatic vibrations are observed at 1348, 1312, and 1266 cm^–1^. The two intense sharp peaks at 830 and 800 cm^–^^1^ are associated with the C–N–C out-of-plane bending of the triazine group. These vibrational spectra can be observed in both **pOMe2CN** and **pOMeAni**. Notably, the additional vibrational mode at 2216 cm^–^^1^, which is associated with the vibration of C≡N, is observed in **pOMe2CN**, indicating successful attachment of the nitrile group.

**Scheme 1:**
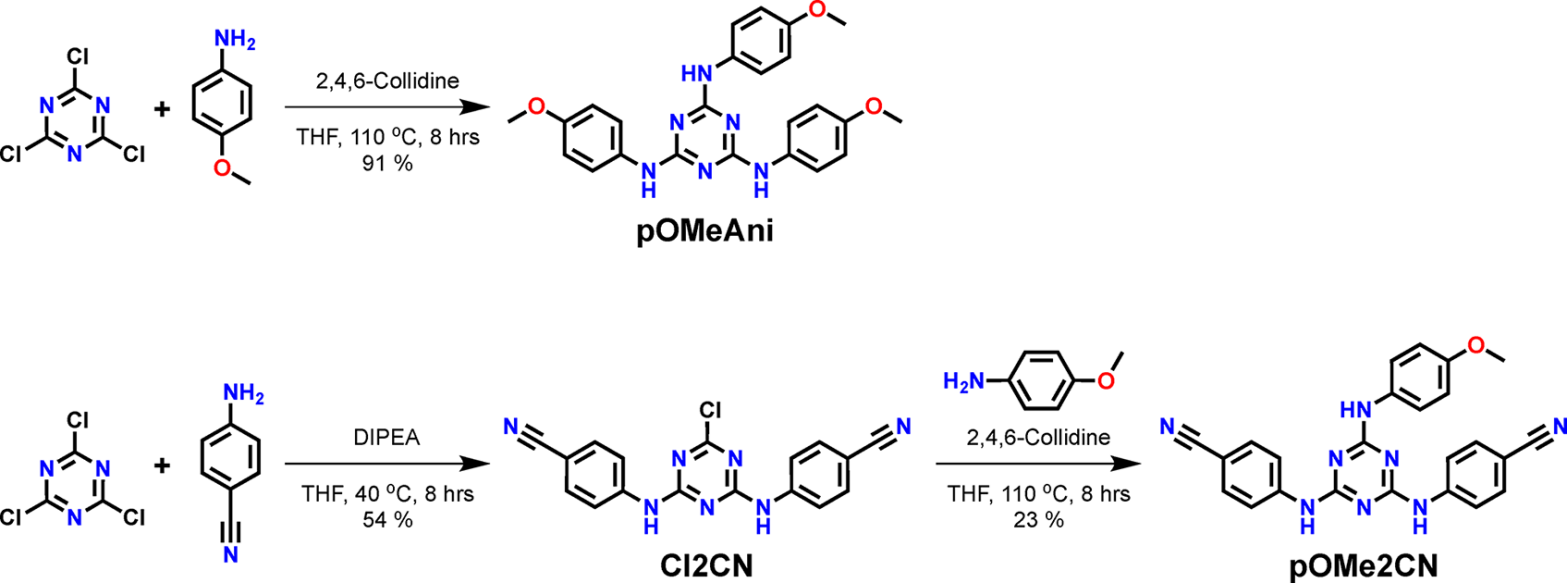
Synthetic Schemes for **pOMeAni** and **pOMe2CN**

**Figure 1:**
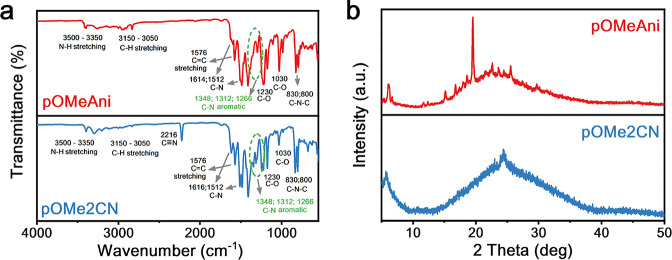
Characterization and optical images of **pOMeAni** and **pOMe2CN**: (a) FT-IR and (b) XRD spectra.

Figures S1 and S2 illustrate the ^1^H and ^13^C NMR spectra of **pOMe2CN** and **pOMeAni**, respectively, which agree well with the proposed molecular structures. The MALDI-TOF MS spectra summarized in Figure S5 show not only a high accuracy with the predicted structure but also show perfect consistency with the theoretically predicted ones. In **pOMeAni**, three *para*-sites of the phenyl ring were functionalized with methoxy (−OCH_3_) groups, and its powder appears orangish and powdery. In contrast, by replacing two methoxy groups with nitrile (−C≡N) groups, **pOMe2CN** shows a white and flaky appearance. Meanwhile, solubility tests were carried out to ensure that the cast materials would not dissolve or peel off in the electrolyte. In this measurement, each active material was soaked in the electrolyte (1 M LiPF_6_ in EC/DEC 1:1 (v/v)) for 7 days. As shown in Figure S6, the UV–vis spectra indicate their excellent structural stability in the electrolyte, as compared with a similar concentration (2.5 uM in a common organic solvent) in battery cells. Furthermore, the powder X-ray diffraction (XRD) spectra (Figure 1b) were obtained to determine their crystallinity. In both XRD spectra, a rough and broad peak was observed at around 25°, which indicates the amorphous structures of triazine-based compounds at room temperature. Notably, **pOMeAni** shows a relatively higher crystallinity than **pOMe2CN** (Figure 1b). This distinct feature could be ascribed to nitrile (−C≡N) group functionalization on the para-sites, which later tuned the packing arrangement of triazine-based small molecules to have a more amorphous structure. This highly amorphous structure could be beneficial to enhancing Li^+^ transport pathways. All spectra are in good agreement with the structure of the materials.

### Electrochemical Performance of Triazine-Based Anodes

This study introduces two triazine-based compounds as LIB anode materials. Initially, half-cells with 40% active materials were fabricated to assess their electrochemical performance, representing a high loading appropriate for realistic device application. Cyclic voltammetry (CV) was performed from 0.02 to 3.0 V (vs Li/Li^+^) at a 0.1 mV s^–^^1^ scan rate to investigate the redox potential. Their fourth cyclic CV curves are shown in Figure S7a (40% **pOMeAni**) and Figure S7b (40% **pOMe2CN**). As shown in Figure S7a, two reduction peaks appeared at 0.90 and 0.02 V, with oxidation peaks at 2.43, 0.95, and 0.12 V. The redox couples at 0.90/0.95 V could be ascribed to the lithiation–delithiation on the nitrogen atoms and C=N in the triazine structure. Meanwhile, redox peaks at 0.02/0.12 V represent Li^+^ insertion and deinsertion in organic moieties (benzene rings). Although the lithiation of the secondary amine is hardly observed at ∼1.9 V, the delithiation process is monitored at 2.43 V. Furthermore, Figure S7c,d shows the Galvanostatic curve of 40% **pOMeAni** and **pOMe2CN** at 100 mA g^–1^ current density, respectively. As depicted in Figure S7c, two low-profile plateaus can be observed at ∼0.9 and 0.02 V on the galvanostatic profile of the **pOMeAni** anode during the charge process, indicating a successful lithiation process, and it is consistent with the CV curves. In addition, **pOMe2CN** showed a similar CV behavior, with the reduction peaks at 0.91 and 0.02 V and oxidation peaks at 2.37, 0.96, and 0.12 V in the CV curve (Figure S7b), suggesting a successful lithiation–delithiation process on the **pOMe2CN** structure. Notably, although **pOMe2CN** has two additional nitrile groups within the structure, no noticeable difference can be observed in the CV curve (Figure S7b) and galvanostatic profile (Figure S7d). This indicates that the nitrile substitution on the triazine-based does not significantly impact the electrochemical behavior; however, it significantly improves the Li^+^ storage capability of **pOMe2CN** (Figure S7d) by providing additional Li-binding sites via nitrile groups.

Furthermore, the maximum capacities of 40% **pOMeAni** and 40% **pOMe2CN** were observed to be 104 and 129 mA h g^–1^, respectively, under 100 mA g^–1^ current density after 100 cycles (Figure S8a). Moreover, rate capability tests were conducted at 100–5000 mA g^–1^ current densities and were switched back to 100 mA g^–1^ to assess the rate capability of the triazine-based electrode (Figure S8b). As depicted in Figure S8b, 40% **pOMeAni** delivered reversible capacities of 100, 92, 82, 71, 60, and 46 mA h g^–1^ at current densities of 100, 200, 500, 1000, 2000, and 5000 mA g^–1^, respectively. Meanwhile 40% **pOMe2CN** delivered reversible capacities of 117, 107, 90, 76, 63, and 48 mA h g^–1^ at current densities of 100, 200, 500, 1000, 2000, and 5000 mA g^–1^, respectively. Furthermore, when the current density was switched back to 100 mA g^–1^, specific capacities of 101 and 119 mA h g^–1^ were recovered for **pOMeAni** and **pOMe2CN**, respectively, demonstrating excellent rate capability. Furthermore, the long-cycle tests at 2000 mA g^–1^, shown in Figure S8c, confirmed structural stability over 2000 cycles, reinforcing the rate capability findings.

To further evaluate the intrinsic material performance, we attempted to reduce active materials to 20%. The reduction of active materials, along with the increase in conductive carbon to 60%, is aimed to increase the conductivity of the electrode and thus minimize the common low conductivity of organic-based electrode materials. The CV curves of 20% anodes are depicted in Figure 2a,b. There was an obvious peak at 0.75 V that can be observed only in the first cathodic scan (Figure 2b, shown in black). The peak was generated due to the formation of an SEI layer at the electrode–electrolyte interface. Notably, a distinct CV behavior is monitored for **pOMe2CN**, where the current density remains low at the first discharge stage before gradually increasing at the next cycle (Figure 2b). This phenomena possibly resulted from higher SEI formation of **pOMe2CN** at the first cycle, thus limiting the initial delithiation kinetics. The higher SEI formation of **pOMe2CN** could have originated from the higher electron exposure due to nitrile groups, thus enhancing the reduction of the electrolyte.^25^ Furthermore, the CV curves of 20% active materials show a reversible redox potential and correspond to those of the 40% anodes with no significant changes during 4 cycles, indicating the structural integrity of the triazine-based anode during the electrochemical process.

**Figure 2:**
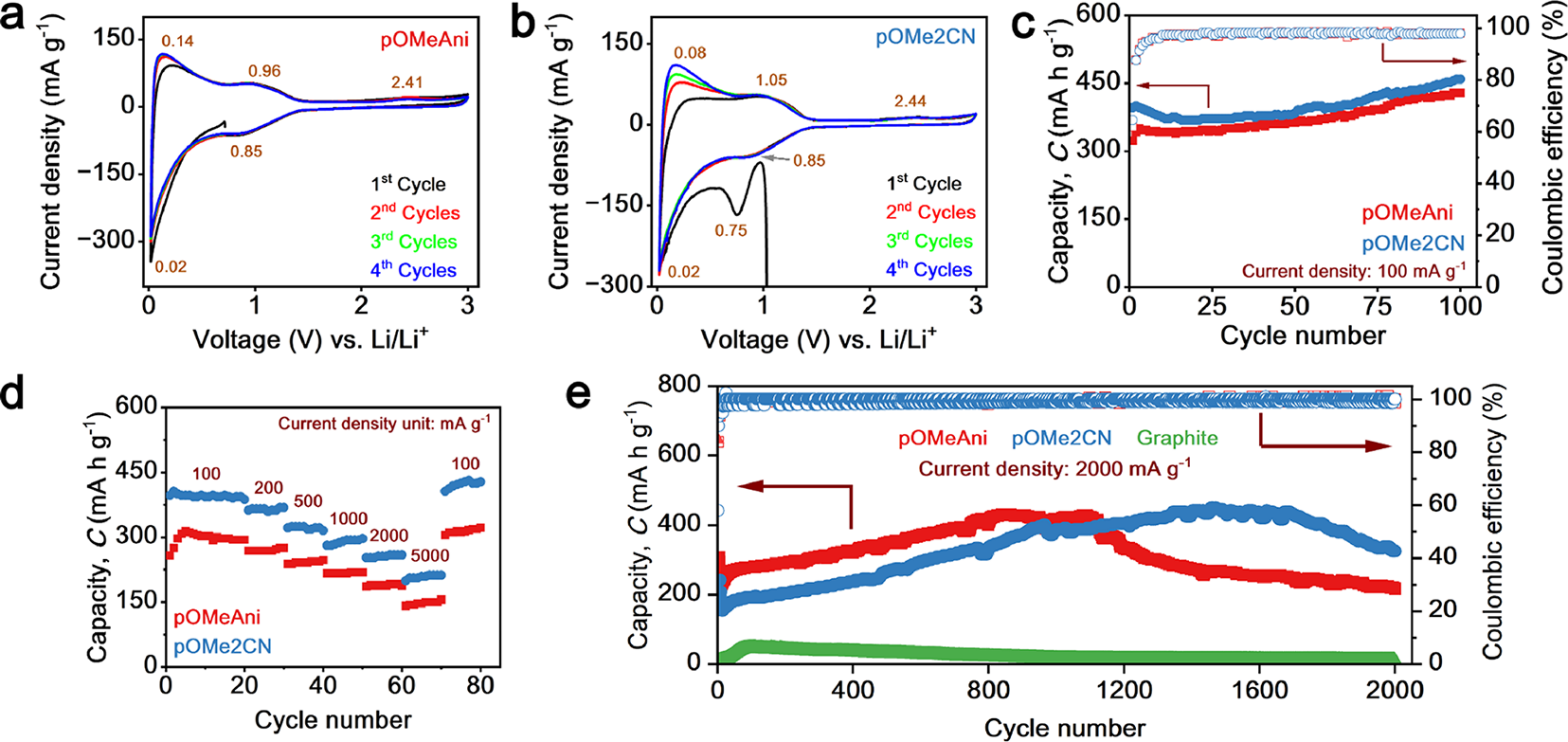
(a) Electrochemical performances of 20% **pOMeAni** and 20% **pOMe2CN**; (b) CV curves; (c) capacity profile at 100 mA g^–1^ current density; (d) rate performance; and (e) long-cycling at 2000 mA g^–1^ current density for 2000 cycles, with 80% graphite as the reference.

To investigate the Li^+^ storage capability, half-cell LIBs consisting of 20% active materials were again prepared and charged and discharged galvanostatically at 100 mA g^–1^ current density with a voltage window of 0.02–3.0 V against Li/Li^+^. Figure S9a,b shows the charge/discharge profiles of 20% **pOMeAni** and 20% **pOMe2CN** anodes over 100 cycles, respectively. During the first lithiation, small irreversible plateaus at 0.85 and 0.93 V were observed, attributed to SEI formation from electrolyte decomposition. The significant drop in specific capacity between the first and second cycles was due to SEI layer formation. Furthermore, the galvanostatic profiles of both 20% **pOMeAni** and 20% **pOMe2CN** anodes were consistent with the CV curve (Figure 2a,b). Notably, the specific capacities of the 20% **pOMeAni** and 20% **pOMe2CN** anodes were significantly increased from 352 and 384 mA h g^–1^ (10th cycle) to 437 and 468 mA h g^–1^ (100th cycle) at 100 mA g^–1^ current density with nearly 99% Coulombic efficiency (CE) (Figure 2c), respectively, nearly three times higher than that of 40% active materials (Figure S8a). These increases in Li-storage capacity could be associated with the increasingly conductive environment and the subsequently accelerated Li-ion transfer. As shown in Figure 2d, both 20% anodes again displayed excellent rate capabilities, with the 20% **pOMeAni** and 20% **pOMe2CN** anodes delivering reversible capacities at various current densities, recovering high capacities of 322 and 428 mA h g^–1^, respectively, when the current density is returned to 100 mA g^–1^. The rate capability of the 20% anodes showed that **pOMe2CN** consistently outperformed **pOMeAni** at all current densities, indicating the better electrochemical performance of 20% **pOMe2CN.**

To assess the long-term durability, cycling tests were performed under a 2000 mA g^–1^ current density for 2000 cycles, including a commercial graphite anode for comparison. As shown in Figure 2e, the 20% **pOMeAni** and 20% **pOMe2CN** anodes exhibited a steady increase in capacity during cycling, reaching peak reversible capacities of 432 mA h g^–1^ at the 865th cycle and 445 mA h g^–1^ at the 1489th cycle, respectively. These performances exceeded that of the commercial graphite anode, where its capacity dropped below 50 mA h g^–1^ after 800 cycles (Figure 2e). Importantly, different cycling behaviors can be monitored under long-term operation. As shown in Figure 2e, **pOMe2CN** exhibits a longer activation period compared to the **pOMeAni** anode. However, **pOMe2CN** reaches the maximum capacity at the 1489th cycle, while **pOMeAni** reaches the maximum capacity at the 865th cycle. The longer activation period observed for **pOMe2CN** compared to that of **pOMeAni** can be attributed to the differences in the gradual formation of a stable SEI during initial cycles. As depicted in Figure 2b, nitrile functional groups in **pOMe2CN** influence the kinetics of SEI formation due to their higher electron exposure characteristics, affecting electrolyte decomposition and resulting in slower electrode activation during the initial cycle. Moreover, this delayed activation period can also be associated with the nature of the −CN electron-withdrawing character that prolonged the activation period due to delocalized electrons, ultimately leading to improved but delayed utilization of active sites.^26,27^ Additionally, gradual capacity fading is observed for both **pOMeAni** and **pOMe2CN** anodes after they reach the maximum capacity (Figure 2e). This capacity fading could result from progressive structural degradation, instability, or continuous thickening of the SEI layer and partial dissolution of the active material during repeated lithiation/delithiation under ultralong cycling processes, as commonly observed in organic electrode materials.^13,28,29^ These notable electrochemical performance of both **pOMeAni** and **pOMe2CN** are comparable among the reported organic-based anodes for LIBs (Table S1). In brief, these electrochemical tests revealed two key findings: (1) lowering the active material from 40% to 20% improves ion accessibility and the utilization efficiency due to an enhanced conductive environment, leading to better performance. (2) The superior overall performance of **pOMe2CN** compared to that of **pOMeAni** could be ascribed to the nitrile groups attached to the **pOMe2CN** structure, which provide additional active sites for Li^+^ storage, thus increasing the capacity and overall performance.

### Li-Ion Storage Mechanism in Triazine-Based Anodes

To probe the Li^+^ storage mechanism in triazine-based anodes, we performed ex situ FT-IR spectroscopy to analyze the changes in the functional groups at different stages (Figure 3). Here, the 40% electrodes were used to obtain the FT-IR spectra. As presented in Figure 3a, when 40% **pOMeAni** was fully lithiated (0.02 V; purple line), the vibrational spectra at 1348, 1312, and 1286 cm^–1^ representing the C–N aromatic stretching in the triazine backbone disappeared and the signals attributed to the C=C aromatic stretching at 1560 cm^–1^ were reduced. The spectral changes evinced a favorable lithiation process in the functional groups of the **pOMeAni** anode. After the 40% **pOMeAni** anode was delithiated at 3.0 V (brown line), the vibrational spectra of C=C and C–N aromatic stretchings re-emerged in the FT-IR spectra (Figure 3a), suggesting a successful delithiation process. Likewise, the FT-IR of 40% **pOMe2CN** at different stages were also presented (Figure 3b). As depicted in Figure 3b, the Li^+^ behavior in the lithiation and delithiation processes were relatively the same as those of **pOMeAni**. As shown in Figure 3b, the vibrational spectra of the C=C and C–N aromatic stretchings were reduced during the lithiation process and were recovered during the delithiation process. Importantly, the additional vibrational spectra of nitrile groups (C≡N) at 2216 cm^–1^ vanished during the lithiation process and reappeared after **pOMe2CN** was fully delithiated. This demonstrates that the additional nitrile groups work as additional Li-binding sites, thus improving the Li-storage capability of **pOMe2CN** compared to that of **pOMeAni**. In addition, a new peak at 1780 cm^–1^ was detected in both spectra at the lithiation and delithiation stages, which are perhaps attributed to carbonate anions (CO_3_^2–^), suggesting the successful formation of SEI layers on the surfaces of the triazine-based anodes during the lithiation/delithiation process. This observation can provide further information related to the Li-storage mechanism in the triazine-based anode during the electrochemical process.

**Figure 3:**
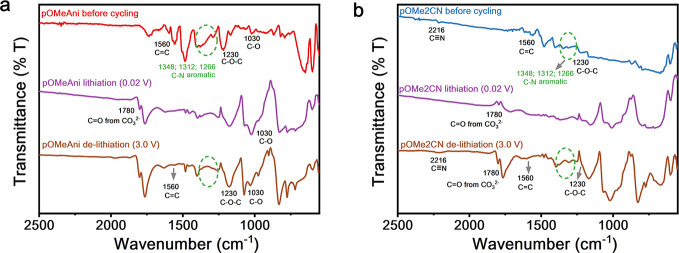
Ex situ FT-IR spectra of 40% triazine-based anodes at different stages: (a) **pOMeAni** and (b) **pOMe2CN**.

With the results obtained from the ex situ FT-IR spectra, we further illustrates the Li^+^ storage mechanism during the lithiation/delithiation process. There are three kinds of active sites (amino, phenyl, and triazine groups) in **pOMeAni**, while there are four kinds of active sites in **pOMe2CN**, with an additional active nitrile group. Based on these assumptions, the possible Li^+^ storage is proposed and presented in Scheme 2. As shown in Scheme 2a, the Li^+^ attach onto N-sites of amino and triazine groups of **pOMeAni**, as well as the phenyl group, during the lithiation process. Meanwhile, additional Li^+^ insertion on N-sites of the nitrile group is monitored, along with Li^+^ attached to the amino and triazine groups and the phenyl group in **pOMe2CN** during the lithiation process. When the delithiation process occurs, the reaction proceeds in a reverse direction associated with the delithiation process. Therefore, based on the aforementioned assumption and the Faraday law, the theoretical capacity of triazine-based anodes can be estimated by the equation presented below:^30–32^

$$C_{T}=nF/3.6M_{W}$$

where *C*_T_ is the theoretical capacity, *n* is the number of Li ions transferred, *F* is the Faraday constant (96,485 C mol^–1^), and *M*_W_ symbolizes the molecular weight. As demonstrated in Scheme 2a, if we presume that the lone pair of electrons on N atoms and the π electron in phenyl groups could serve as Li^+^ binding sites, **pOMeAni** (*M*_w_ = 444.49 g mol^–1^) can hold approximately 14 Li ions in its structure, which is attributed to Li^+^ insertion in the triazine (5 Li^+^), phenyl (6 Li^+^), and amino (3 Li^+^) groups.^20^ These 14 Li ions in each **pOMeAni** compound generates a theoretical capacity of approximately 844.16 mA h g^–1^. Similarly, **pOMe2CN** (*M*_w_ = 434.45 g mol^–1^) can accommodate approximately 16 Li ions in the structure, thus corresponding to a theoretical capacity of approximately 987.05 mA h g^–1^. This indicates that additional nitrile groups significantly enhance the theoretical capacity of the triazine-based anode. The increasing theoretical capacity of **pOMe2CN** due to additional nitrogen atoms by incorporating nitrile groups is in good agreement with previous studies.^26,33–35^ However, there is a constraint between the practical (Figure 2c) and theoretical capacity of the triazine-based anode. This discrepancy could be ascribed to steric hindrance, pore blockage, or limited diffusion paths that result in the inaccessible active sites of the triazine-based anode under practical testing conditions. Moreover, previous studies have also shown that although nitrile groups increase the theoretical capacity, they sometimes have a counter effect by minimizing electron transport at the neighboring active sites, thus limiting the practical capacity.^21^ Besides, the actual capacity measured experimentally also often depends on the surface morphology and SEI formation.^25,36,37^

**Scheme 2:**
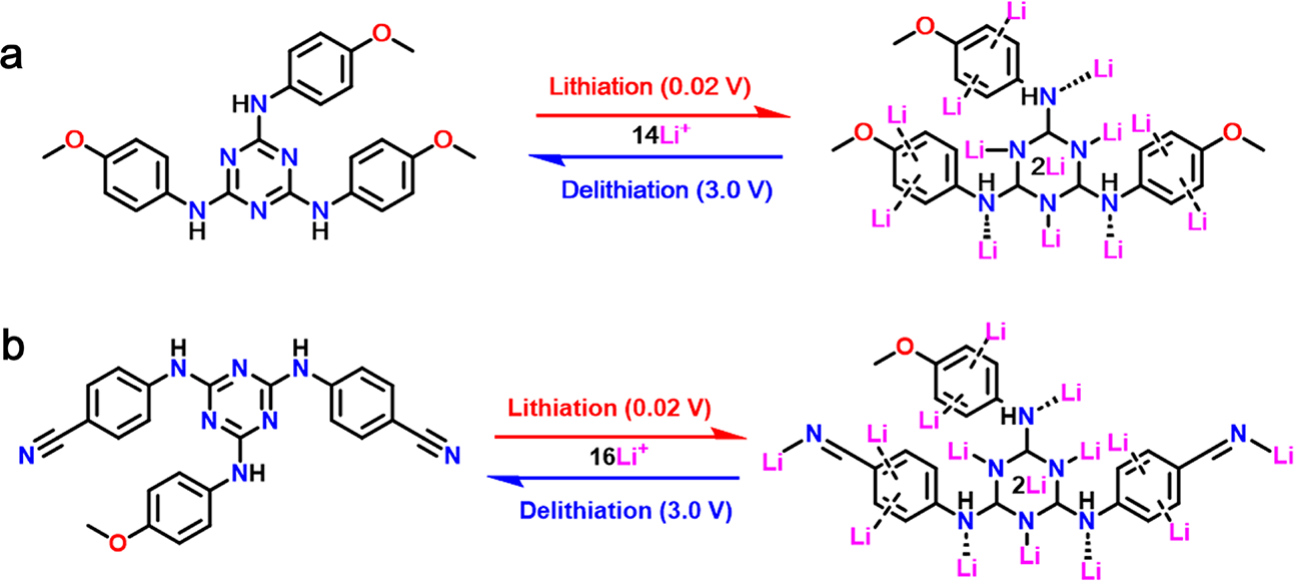
Lithium-Ion Storage Mechanism in Triazine-Based Anodes^a^

a (a) **pOMeAni** and (b) **pOMe2CN**.

### Kinetic Studies of Triazine-Based Anodes

To further gain deep insights into the triazine-based anode during the electrochemical process, we performed charge storage and kinetic studies to assess the chemistry within triazine-based anodes. First, electrochemical impedance spectroscopy (EIS) was performed from 10 mHz to 1 MHz with a 10 mV AC amplitude to assess charge-transfer properties in both 20 and 40% anodes. The EIS analysis is a useful method to study the kinetic phenomena at the interface and Li^+^ transport inside the electrode, which can help figure out the relationship between the loading of active materials and performance. Figure S10 shows Nyquist plots of **pOMeAni** and **pOMe2CN** anodes before and after 100 cycles with one semicircle in all stages. As described in Table 1, the bulk resistance (*R*_s_) related to the overall system resistance (the conduction through the electrolyte, separator, and wires) changed only marginally during the charging/discharging operation, showing that the triazine-based materials were greatly stable during electrochemical cycling. This stability reflects minimal obstruction from electrolytes, separators, and wires during the electrochemical process. Additionally, the charge-transfer resistance (*R*_ct_) in the midfrequency part, which is associated with the Li^+^ conduction across two interfaces during electrochemical operation, the electrode–electrolyte interfaces, shows that both 20% anodes showed a significantly lower resistance than the 40% anodes before and after 100 cycles (Table 1), aligning with their better battery performance. This further confirmed that increasing the conductive environment in the 20% anodes is effective to improve the charge-transfer reaction within the triazine-based anode. Moreover, this EIS observation also highlights that the lower capacity at 40% than at 20% primarily arises from reduced conductive pathways and increased *R*_ct_, an effect of the electrode formulation rather than a dissolution issue of active materials. Notably, the diffusion coefficient (*D*_Li_) of **pOMe2CN** was one order of magnitude higher than that of **pOMeAni** after 100 cycles in both 20 and 40% anodes. This indicates that additional nitrile groups could significantly improve the Li^+^ mobility, thus leading to an improvement of the storage capacity.

**Table 1:** Fitted Data of the EIS Spectra of **pOMeAni** and **pOMe2CN** before Cycling and after 100 Cycles at 100 mA g ^–1^

sample	stages	*R*_s_ [Ω]	*R*_ct_	diffusion coefficient [cm^2^ s^–1^]
20% **pOMeAni**	before cycling	6.187	240.0	1.17 × 10 ^–14^
	after 100 cycles	11.28	151.8	1.28 × 10 ^–12^
40% **pOMeAni**	before cycling	7.727	439.3	6.00 × 10 ^–14^
	after 100 cycles	9.363	321.7	8.80 × 10 ^–15^
20% **pOMe2CN**	before cycling	5.294	144.7	1.95 × 10 ^–13^
	after 100 cycles	8.526	154.5	2.03 × 10 ^–12^
40% **pOMe2CN**	before cycling	6.867	267.8	1.62 × 10 ^–11^
	after 100 cycles	7.821	238.9	1.17 × 10 ^–14^

Furthermore, we performed sweep rate voltammetry at different scan rates in the potential window ranging from 0.02 to 3.0 V (against Li/Li^+^) to estimate the charge storage mechanism of the triazine-based anode (see Figure S11). The sweep rate method provides information on the charge storage behavior of electrode active materials, such as the triazine-based anode during the charge/discharge process. In the sweep rate CV, the total charge storage in a CV can be sorted into three different types: (a) the non-Faradaic charge storage from the physical separation of charges, i.e., the so-called electrical double-layer effects; (b) the Faradaic charge storage from pseudocapacitance, which is a charge storage phenomenon of an electrochemical system that is responsive in nature; and (c) the Faradaic charge storage from the transfer of electrons across an interface, the electrode–electrolyte interface, which is diffusion-limited.^38–41^

Based on the power–law relationship, the charge storage behavior of electrode active materials at different scan rates can be estimated by the following equation:

$$i=av^{b}$$

where *a* and *b* are constants. The CV data can be converted to a plot of log (*i*) vs log (*v*), which expresses their linear relationship and the *b*-value is the slope of the linear fitted line. A b-value close to 0.5 indicates that the system exhibits Faradaic behavior. On the other hand, if the *b*-value were close to 1, a capacitive or pseudocapacitive behavior is dominant. As shown in Figure 4a,b, all of the electrodes demonstrate a reduction in specific current as the scan rate was reduced from 1 to 0.1 mV s^–1^. Attractively, it is noticed that the *b*-values of 40% **pOMe2CN** are slightly higher (20%: 0.94; 40%: 0.89) than those of **pOMeAni** (20%: 0.87; 40%: 0.86). This suggests that replacing nitrile groups in the triazine-based electrode remarkably enhances the extent of capacitive effects. This suggests that the free electrons in the nitrile groups enhance the absorptivity of Li^+^, thus enhancing the pseudocapacitive behavior.

**Figure 4:**
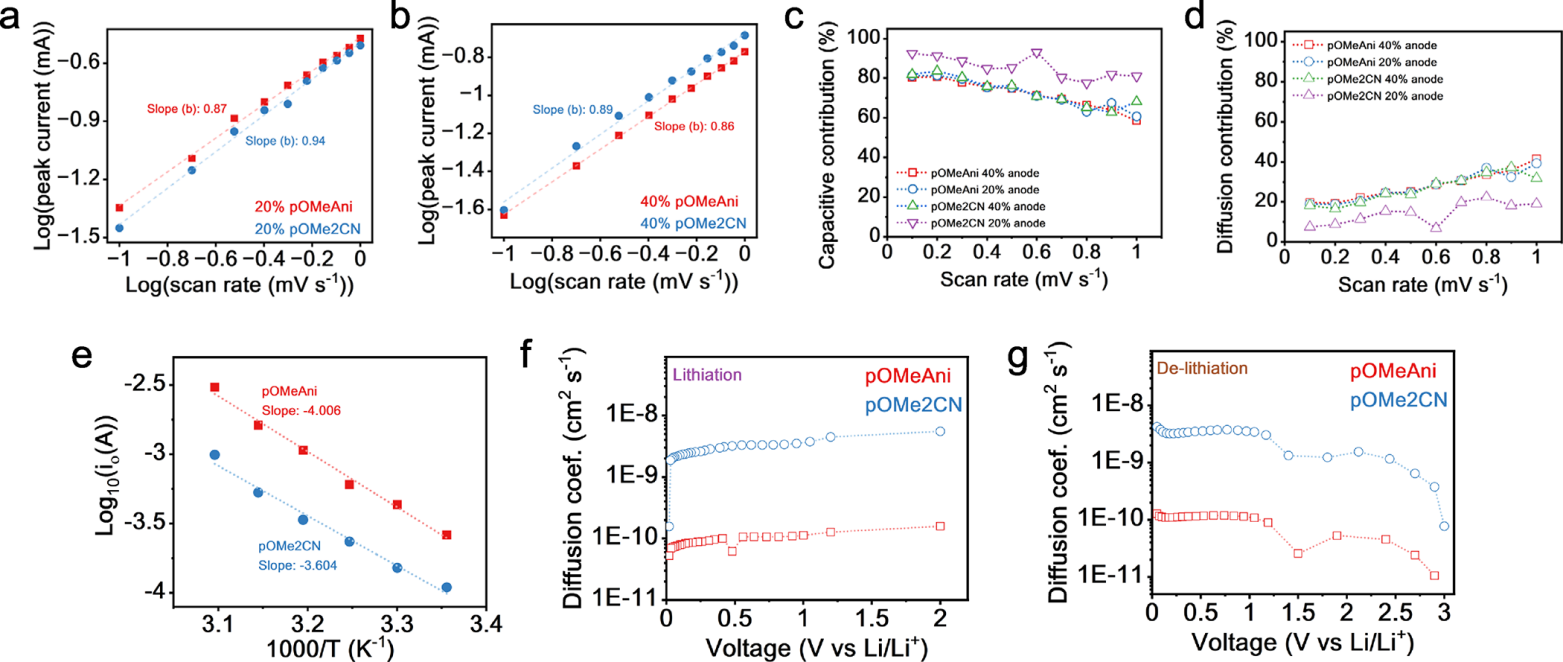
Charge storage mechanism of triazine-based anodes: the log peak current (*i*) against the log scan rate (*v*) plot: (a) 20% and (b) 40%. (c) Capacitive contributions and (d) diffusion contributions at various scan rates. (e) Log *i*_0_ vs 1000/*T* plot. Comparison between the Li ion diffusion coefficients derived from the GITT method: (f) lithiation and (g) delithiation.

In addition, the total charge stored for the capacitive (*k*_1_ *v*) and diffusion (*k*_2_ *v* ^1/2^) contributions at each defined potential can be further estimated by using the following equation:

$$i=k_{1}v+k_{2}v^{1/2}$$

where *k*_1_ and *k*_2_ values can be estimated by plotting *i*/*v* ^1/2^ vs *v* ^1/2^ (Figure S12a,b). The capacitive and diffusion contribution ratios of 20 and 40% of both **pOMeAni** and **pOMe2CN** are presented in Figure 4c,d, respectively. Conforming to the analysis of the *b*-value (Figure 4a,b), both 20% electrodes showed an improved capacitive character compared to the 40% electrodes (Figure 4c). As shown in Figure 4c,d, the charge storage behavior of **pOMeAni** changed only slightly when the content in the electrode is reduced from 40% to 20%, which is consistent with the b-value (Figure 4a,b). Notably, a significant improvement in the capacitive behavior is observed in **pOMe2CN** when the content in the electrode is reduced from 40 to 20%. This indicated that an increasingly conductive environment in the electrode synergistically improves the absorptivity of Li^+^, leading to enhanced storage capability.^42^

Additionally, we evaluated the activation energy (*E*_a_) of the triazine-based anode to gain a deep understanding of the higher battery performance in **pOMe2CN** from a thermodynamic perspective. In brief, the activation energy (*E*_a_) was determined using the EIS method at various temperatures to observe changes in the charge-transfer resistance with temperature (see Figure S13a,b). This technique has been used in some studies in the literature to effectively probe the *E*_a_ of electroactive materials, in which the detailed relationship between the exchange current (*i*_0_), charge-transfer resistance at the electrode interface (*R*_ct_) at various temperatures, and the apparent activation energy (*E*_a_) for Li ions binding to the compounds can be organized into the Arrhenius equation as shown below:^26,43–45^

$$i_{0}=RT/(nFR_{\mathrm{ct}});i_{0}=A\exp(-E_{a}/RT)$$

where *R* is the gas constant (8.314 J K^–1^ mol^–1^), *T* (K) is the absolute temperature, *n* is the number of transferred ions, *F* is the Faraday constant (96,485 C mol^–1^), A is a temperature-independent coefficient, and *E*_a_ is the apparent activation energy. Figure 4e demonstrates the Arrhenius plots of the linear relationship between log_10_ *i*_0_ and 1/*T*. The apparent activation energy is *E*_a_ = −*Rk*ln10, where *k* is the slope of the fitting line. As shown in Figure 4e, the *E*_a_ values of the triazine-based anode were estimated as 76.691 and 68.998 kJ mol^–1^ for **pOMeAni** and **pOMe2CN**, respectively. These results denote that **pOMe2CN** has a significantly lower *E*_a_ than **pOMeAni**, suggesting a higher acquisition ability of acquiring Li ions from host compounds. The observed results are in good agreement with the electrochemical performance displayed in Figures 2c–e and S9.

Lastly, the galvanostatic intermittent titration technique (GITT) was performed to probe the Li ion diffusion abilities during the lithiation and delithiation processes. GITT is a method that applies multiple pulse currents with a specific resting time, which can analyze the relationship between the potential and time to obtain information related to the reaction kinetics.^46,47^ Figure S14a,b shows the galvanostatic profiles of **pOMeAni** and **pOMe2CN** during GITT measurement. As presented in Figure 4f,g, it is clearly seen that the diffusion coefficient of **pOMe2CN** is nearly 2 orders of magnitude higher than that of **pOMeAni** during both lithiation or delithiation processes, indicating that the ion diffusion rate was significantly increased in the **pOMe2CN** anode due to nitrile-group functionalization. In brief, these mechanistic and kinetic studies confirm that the beneficial properties of a more conductive environment and nitrile-group functionalization could significantly improve the electrochemical performance.

## Conclusions

In summary, our study illustrated new small active compounds of various available Li^+^ binding sites in triazine-based electrodes. The triazine, phenyl, amino, and nitrile groups accelerate the amount of effective and reversible Li^+^ storage inside the triazine-based material structures. As a result, the Li^+^ binding sites are activated during the cycling, and more effective Li^+^ storage can be achieved because of the increased area of electrolyte penetration inside the structure of the triazine-based materials. Moreover, compared with the performance of **pOMeAni**, the **pOMe2CN** anode exhibits remarkable excellence, as supported by a series of electrochemical measurements. This work not only added effective active sites by tuning the structure but also lowered the activation energy and increased the diffusion coefficient. All of these advantages imply that this modification is a win-win strategy and provides insights for the next-generation LIBs.

## Experimental Section

### Materials

All reagents were used as received without further purification.

### Syntheses of **pOMe2CN** and **pOMeAni**

At room temperature, 4-aminobenzonitrile (1.19 g, 10.0 mmol) was dissolved in tetrahydrofuran (THF; 25 mL) in a round-bottom flask, followed by the slow addition of cyanuric chloride (0.93 g, 5.0 mmol). The reaction mixture was stirred for 30 min, and *N,N*-diisopropylethylamine (DIPEA; 5.30 mL, 30.4 mmol) was then added drop-wise. The mixture was then stirred for 8 h at 40 °C. The reaction mixture was added to an aqueous potassium carbonate solution (1.04 g, 7.5 mmol) and then extracted with dichloromethane (100 mL). The organic layer was dried over anhydrous magnesium sulfate and filtered. The filtrate was concentrated at reduced pressure to yield the crude product, which was then recrystallized from THF and hexane to afford **Cl2CN** (0.94 g, yield: 54.1%).

**Cl2CN** (0.70 g, 2.0 mmol), 4-methoxyaniline (0.25 g, 2.0 mmol), and 2,4,6-collidine (0.80 mL, 6.0 mmol) were added to a sealed tube and heated at 110 °C for 8 h to give a crude product, which was purified by chromatography [eluate: DCM/THF (4:1)]. **pOMe2CN** (0.20 g, yield: 23.1%) was then obtained.

**pOMeAni** was prepared from the reaction of cyanuric chloride (0.93 g, 5.0 mmol) and 4-methoxyaniline (1.85 g, 15.0 mmol) in the presence of 2,4,6-collidine (6.0 mL, 45.2 mmol) in a sealed tube at 110 °C for 8 h. **pOMeAni** (2.03 g, yield: 91.3%) was isolated after purification by chromatography as described above.

#### pOMe2CN

^**1**^ **H NMR (300 MHz, DMSO-** ***d***_**6**_ **)** δ 9.83 (s, 2H), 9.44 (s, 1H), 8.03–8.06 (m, 4H), 7.71–7.76 (m, 4H), 7.60 (d, *J* = 8.7 Hz, 2H), 6.94 (d, *J* = 8.7 Hz, 2H), 3.76 (s, 3H) (Figure S1). ^**13**^ **C NMR (75 MHz, DMSO-** ***d***_**6**_ **)** δ 164.1, 163.9, 155.3, 144.5, 132.9, 132.1, 123.0, 119.7, 119.5, 113.8, 103.3, 55.3 (Figure S1). **HRMS (ESI-TOF)** *m*/*z*: [M + H]^+^ calcd for C_24_H_19_N_8_O 435.1682, found 435.1676 (Figure S5b). **IR (ATR)**: *ṽ* = 3404, 3290 (N–H stretching), 2955 (C–H asymmetric stretching), 2836 (C–H symmetric stretching), 2224 (C≡N stretching), 1617, 1509 (C=N stretching), 1571 (C=C stretching), 1032 (C–O stretching) cm^–1^ (Figure S3).

#### pOMeAni

**^1^H NMR (300 MHz, DMSO-** ***d***_**6**_ **)** δ 8.96 (s, 3H), 7.63–7.66 (m, 6H), 6.84–6.88 (m, 6H), 3.73 (s, 9H) (Figure S2). ^**13**^ **C NMR (75 MHz, DMSO-** ***d***_**6**_ **)** δ 164.1, 154.6, 133.1, 122.1, 113.5, 55.2 (Figure S2). **HRMS (ESI-TOF)** *m*/*z*: [M + H]^+^ calcd for C_24_H_25_N_6_O_3_ 445.1988, found 445.1983 (Figure S5a). **IR (ATR)**: *ṽ* = 3390 (N–H stretching), 2952 (C–H asymmetric stretching), 2834 (C–H symmetric stretching), 1612, 1512 (C=N stretching), 1578, 1561 (C=C stretching), 1030 (C–O stretching) cm^–1^ (Figure S4).

### Preparation of Triazine-Based Anodes

To compare the performance of different recipes, the anode slurries were prepared with two different active material ratios: 20 and 40%. The mixtures were prepared by mixing 20% (40%) as-synthesized trazine-based **pOMeAni** or **pOMe2CN**, 60% (40%) conductive carbon (Super P; > 99% (metal basis); UBIQ Technology Co. Ltd.), poly­(vinylidene fluoride) (PVDF; UBIQ Technology Co. Ltd.) as a binder, and *N*-methyl-2-pyrrolidone (NMP; Thermo Fisher Scientific) as a solvent. Then, these mixtures were stirred for 24h to obtain a homogeneous slurry. The slurry was cast onto Cu foil and dried on a hot plate at 60 °C overnight; then, the foil was dried at 80 °C for 8 h in an oven under vacuum. For reference, a slurry consisting of 80% graphite (natural graphite powder (GN-580L); UBIQ Technology Co. Ltd.), 10% Super P, 10% PVDF, and NMP as a solvent was also prepared. The 20 and 40% **pOMeAni**, 20% and 40% **pOMe2CN**, and 80% graphite electrodes were cut into small disks of 12 mm diameter, with average loadings of 2.43 (20%), 4.85 (40%), and 2.04 mg cm^–1^, respectively, before being placed in a glove box for the coin cell assembling process.

### Coin Cell Assembly and Electrochemical Tests

For battery assembly, we use a CR2032 type coin cell. The coin cells were fabricated in a high-purity Ar-filled glove box (Vigor, Vigor Tech USA; H_2_O *<* 0.5 ppm, O_2_ *<* 0.5 ppm) using the as-prepared triazine-based anode as a working electrode, Li-metal foil as a counter/reference electrode, Celgard 2325 as a separator, and 40 μL of 1 M LiPF_6_ in 1:1 (v/v) ethylene carbonate/diethyl carbonate (EC/DEC; UBIQ Technology Co. Ltd.) as the electrolyte. The charge and discharge galvanostatic tests were operated on AcuTech battery station systems (AcuTech Systems Co. Ltd.). CV was performed on a PalmSens BV within a voltage window of 0.02–3.0 V. EIS was performed before and after battery cycling on a CHI electrochemical workstation model 760e, CH Instruments Inc. The EIS measurement was obtained with an alternating current (AC) voltage signal of 10 mV and over a frequency range of 1 MHz–0.01 Hz, and the CV measurements were performed at a scan rate of 0.1 mV s^–1^. The GITT was performed using a potentiostat (Squidstat; Admiral Instruments) with the current pulse for 20 and 10 min resting periods at 50 mA g^–1^ current density.

### Material Characterization

^1^H and ^13^C NMR spectra were recorded in a solution on a Bruker AMX-300 NMR spectrometer. Matrix-assisted laser desorption ionization time-of-flight (MALDI-TOF) mass spectrometry was performed on a Bruker Autoflex Speed TOF/TOF spectrometer. Infrared (IR) spectra were obtained by using a PerkinElmer Frontier FT-IR spectrometer equipped with an ATR accessory.

## Supplementary Material



## Notes

NMR spectra, FT-IR spectra, MALDI-TOF mass spectra, solubility test, cyclic voltammograms, galvanostatic profiles, cycling performance, FESEM images, EIS spectra, and GITT curves (PDF)
