# Supplementary material for: Functionalized Triazine-Based Small Molecules as Efficient Battery Anode Materials
Source: ACS Omega. 2025 Sep 16;10(38):43940–9. doi: 10.1021/acsomega.5c04787 (PMC12489688; doi:10.1021/acsomega.5c04787)
Supplement: Supplementary file 1 [file ao5c04787_si_001.pdf]

Supporting Information

Functionalized Triazine-based Small Molecules as Efficient Battery Anode Materials

Yao-Chih Lu[1,||], Febri Baskoro[2,||], Meng-Ju Yang[3,||], Hung-Ju Yen[3,] and Long-Li Lai[1,*]*

[1] *Department of Applied Chemistry, National Chi Nan University, No. 1 University Rd., Puli, Nantou 545, Taiwan.*

[2] *Material Science and Engineering Research Group, Faculty of Mechanical and Aerospace Engineering, Institut Teknologi Bandung, Jl. Ganesha 10, Bandung 40132, Indonesia.*

[3] *Institute of Chemistry, Academia Sinica, 128 Academia Road, Section 2, Nankang, Taipei 11529, Taiwan.*

[||] These authors contributed equally.

E-mail: hjyen@gate.sinica.edu.tw (H. J. Yen); lilai@ncnu.edu.tw (L.L. Lai)

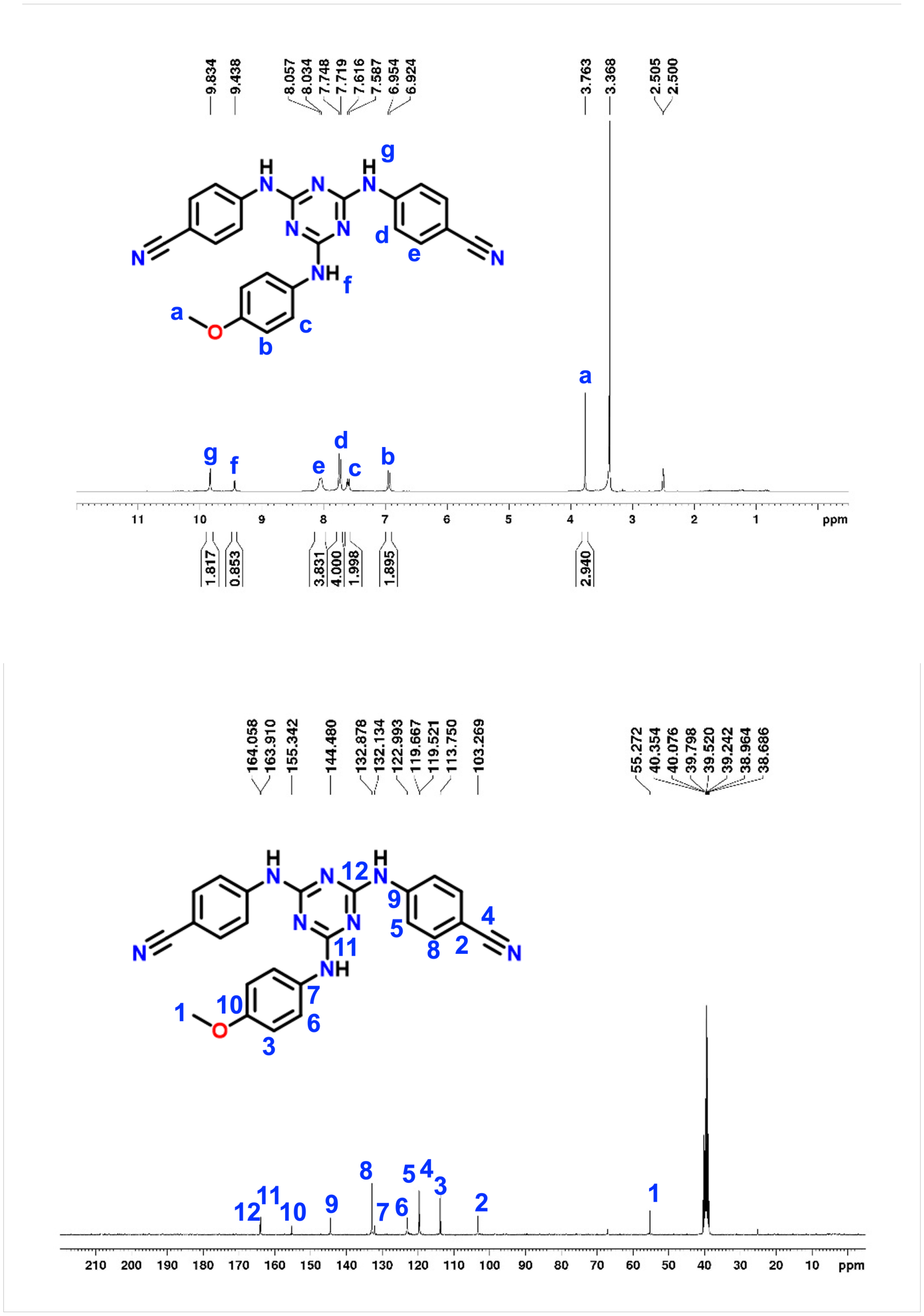

Figure S1. ^{1}H and ^{13}C NMR spectra of **pOMe2CN**.

^{1}H NMR (300 MHz, DMSO-d$_6$) δ 9.83 (s, 2H), 9.44 (s, 1H), 8.03-8.06 (m, 4H), 7.71-7.76 (m, 4H), 7.60 (d, J = 8.7 Hz, 2H), 6.94 (d, J = 8.7 Hz, 2H), 3.76 (s, 3H).

^{13}C NMR (75 MHz, DMSO-d$_6$) δ 164.1, 163.9, 155.3, 144.5, 132.9, 132.1, 123.0, 119.7, 119.5, 113.8, 103.3, 55.3.

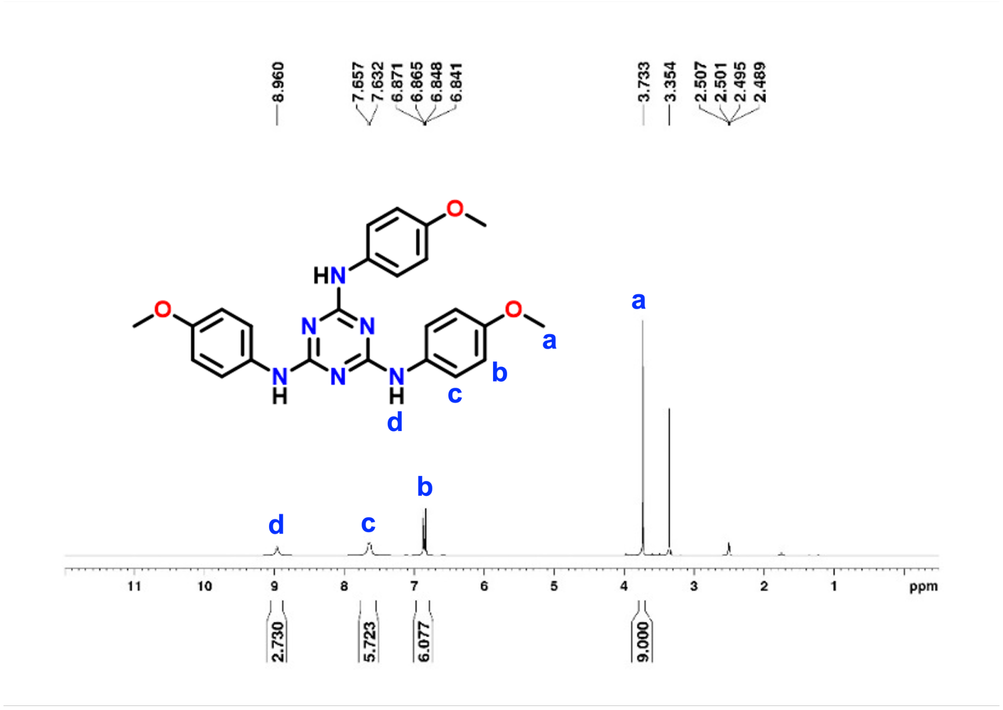

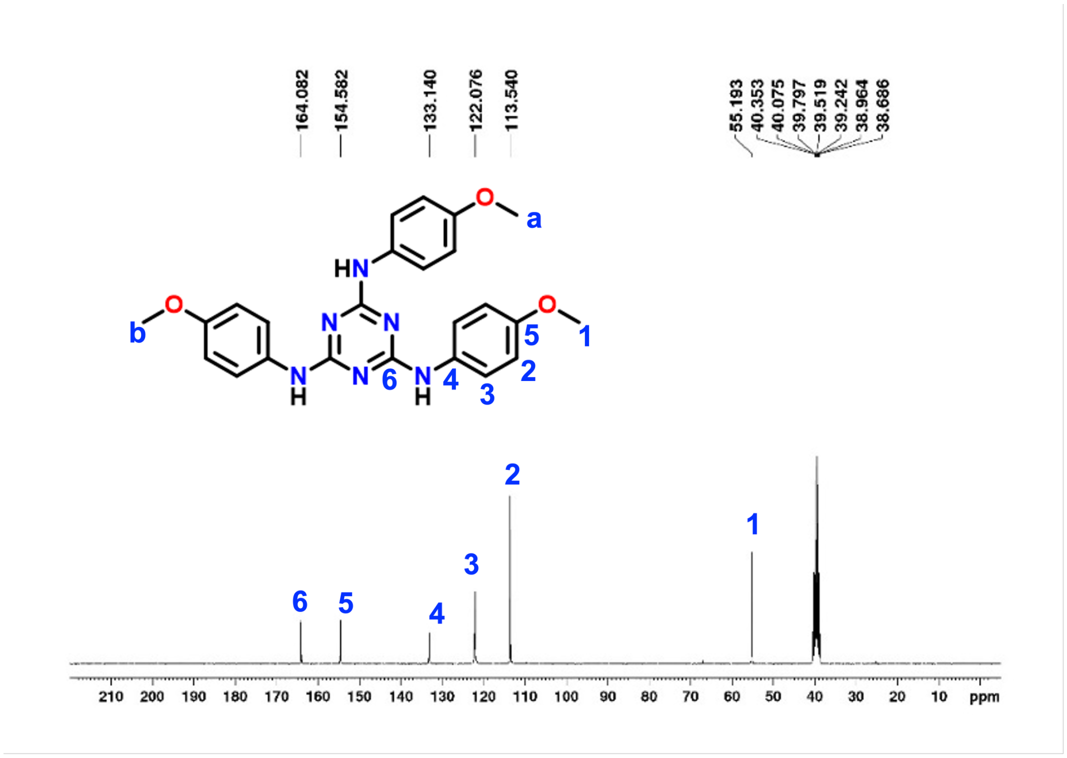

Figure S2. ^{1}H and ^{13}C NMR spectra of **pOMeAni**.

^{1}H NMR (300 MHz, DMSO-d_6) δ 8.96 (s, 3H), 7.63-7.66 (m, 6H), 6.84-6.88 (m, 6H), 3.73 (s, 9H).

^{13}C NMR (75 MHz, DMSO-d_6) δ 164.1, 154.6, 133.1, 122.1, 113.5, 55.2.

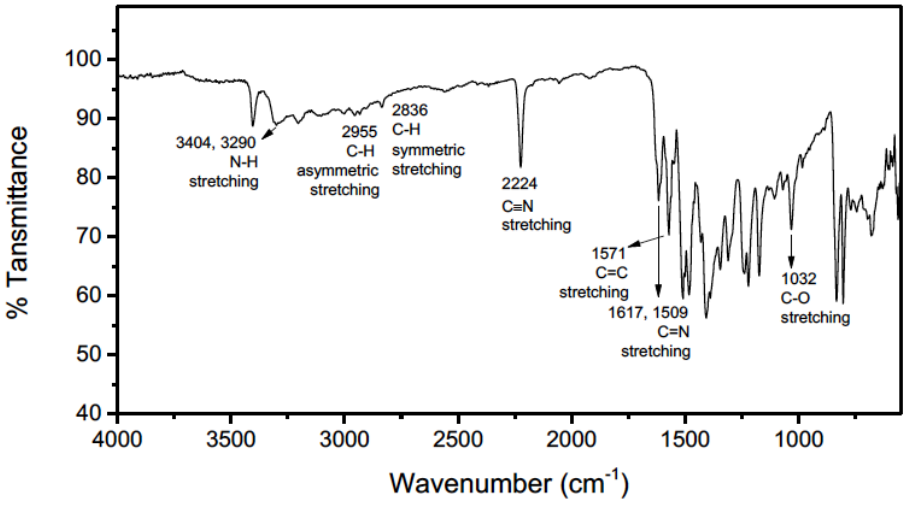

Figure S3. FT-IR spectrum of **pOMe2CN**.

IR (ATR): $\tilde{v}$ = 3404, 3290 (N–H stretching), 2955 (C–H asymmetric stretching), 2836 (C–H symmetric stretching), 2224 (C≡N stretching), 1617, 1509 (C=N stretching), 1571 (C=C stretching), 1032 (C–O stretching) cm^{-1}

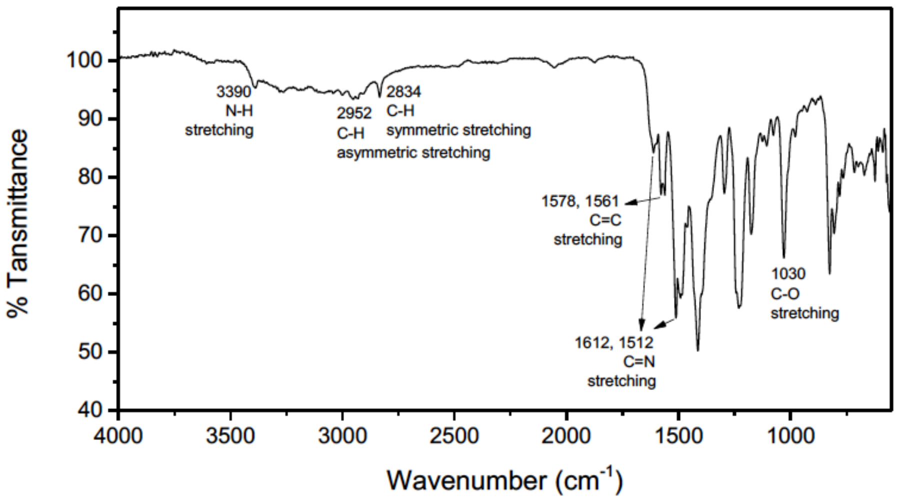

Figure S4. FT-IR spectrum of **pOMeAni**.

IR (ATR): $\tilde{\nu}$ = 3390 (N–H stretching), 2952 (C–H asymmetric stretching), 2834 (C–H symmetric stretching), 1612, 1512 (C=N stretching), 1578, 1561 (C=C stretching), 1030 (C–O stretching) cm^{-1}

(a)

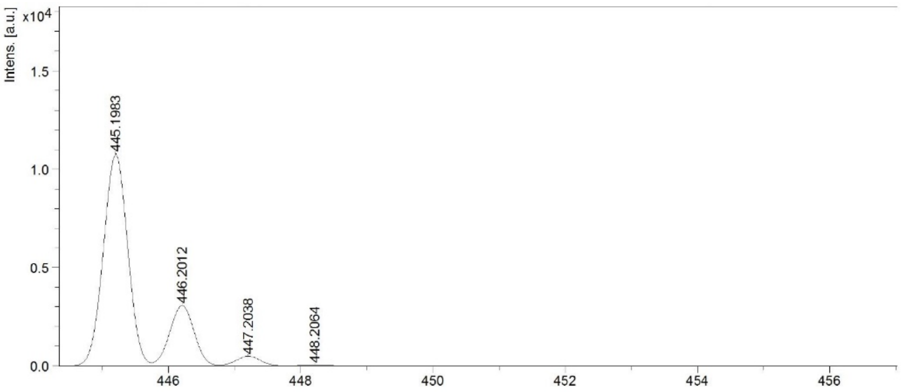

(b)

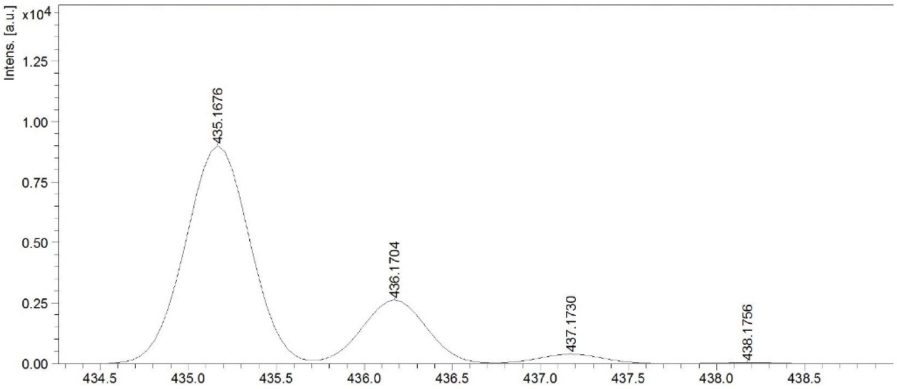

Figure S5. HRMS (MALDI-TOF). (a) **pOMeAni**, m/z: $[M + H]^+$ calcd for $C_{24}H_{25}N_6O_3$ 445.1988, found 445.1983. (b) **pOMe2CN**, $[M + H]^+$ calcd for $C_{24}H_{19}N_8O$ 435.1682, found 435.1676.

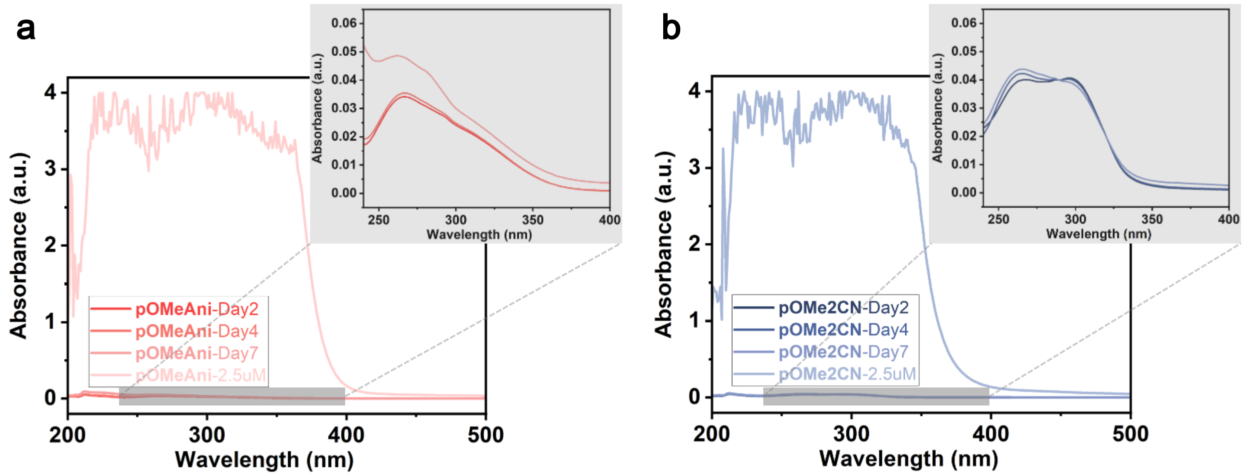

Figure S6. Solubility tests, soaking (a) **pOMeAni**; (b) **pOMe2CN** in 1 M $LiPF_6$ in EC:DEC (1:1 v/v) for 2, 4, and 7 days, as compared with the similar concentration (2.5 uM in common organic solvent) in battery cell.

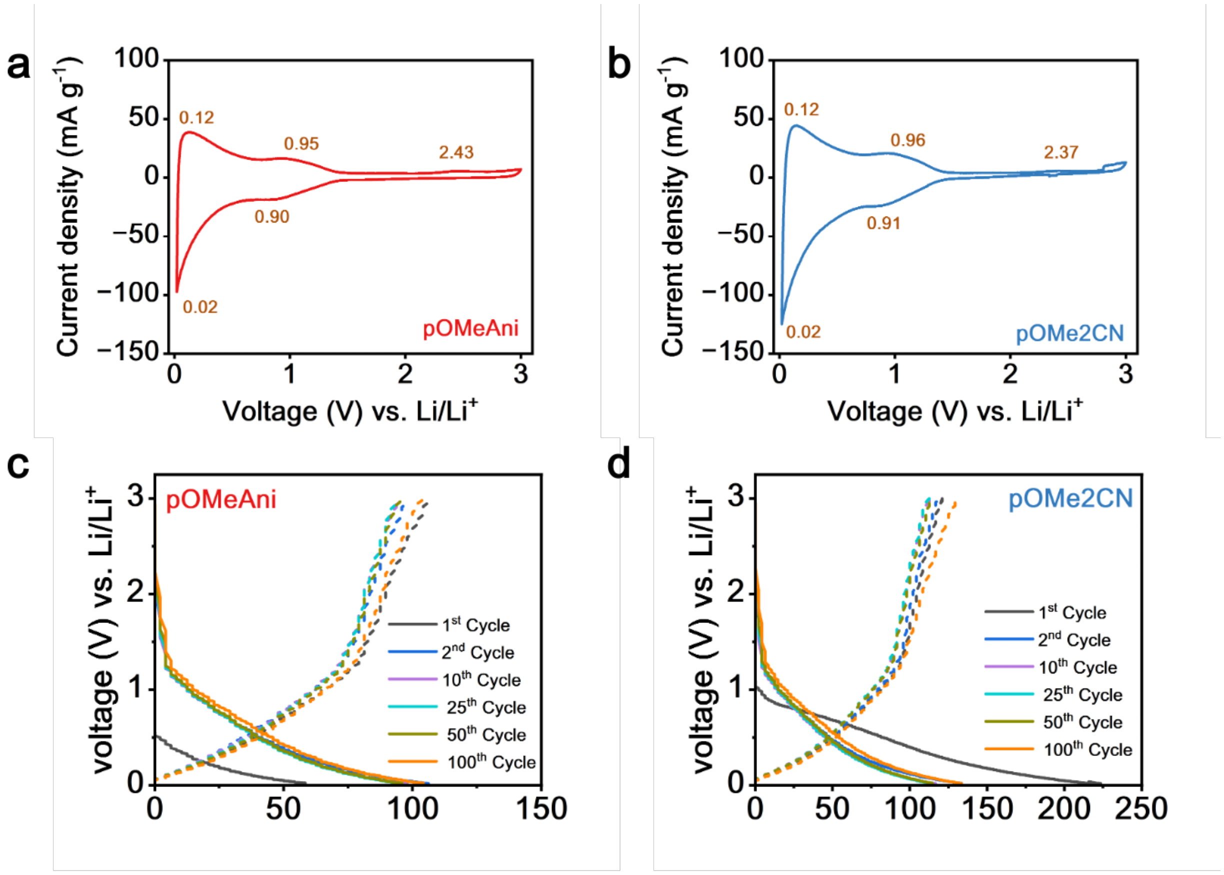

Figure S7. (a – b) the 4th cycle of cyclic voltammetry (CV) and (c – d) Galvanostatic charge-discharge curves at 100 mA g^{-1} of 40% anodes **pOMeAni** and **pOMe2CN**, respectively.

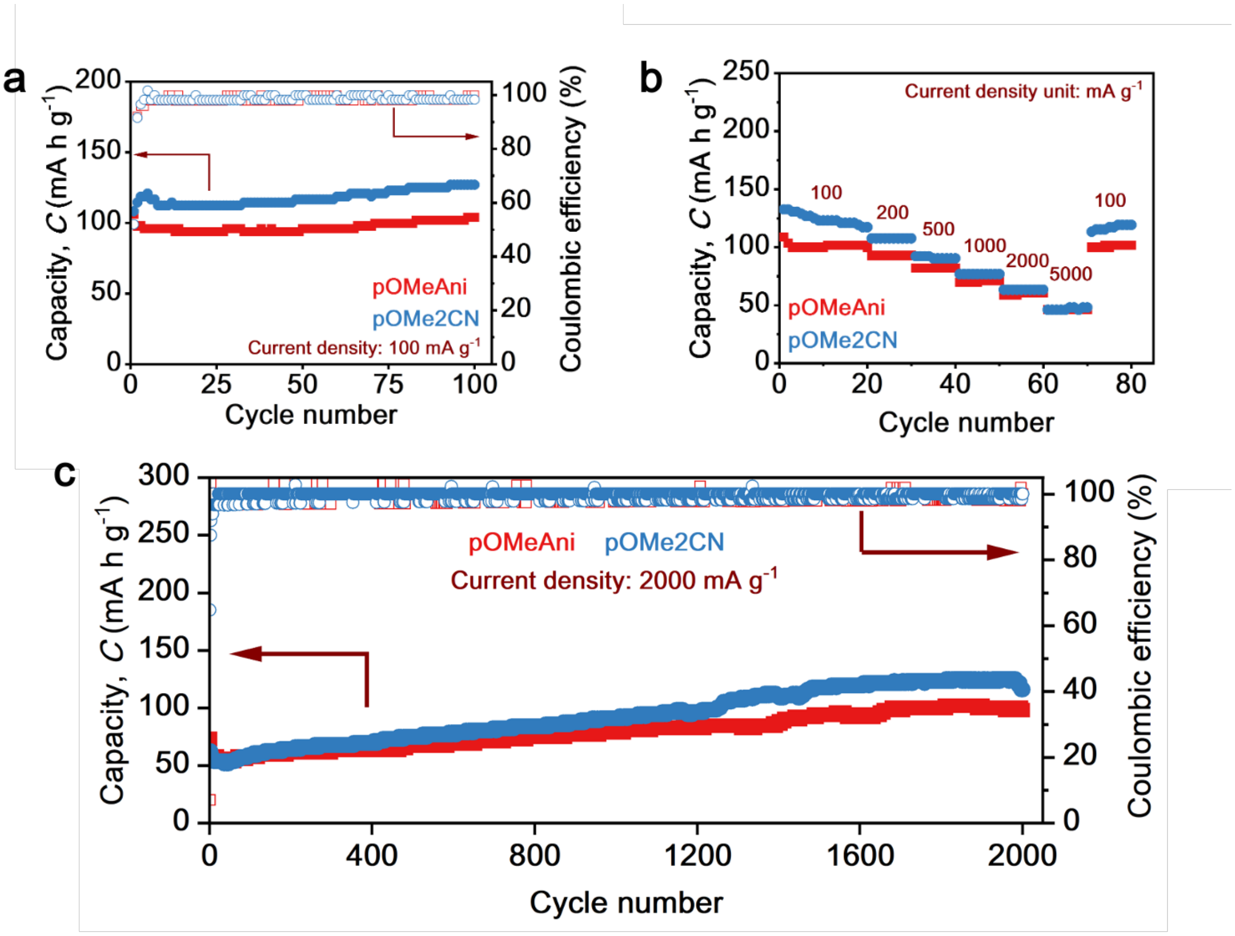

Figure S8. Electrochemical performance of 40% **pOMeAni** and **pOMe2CN** (a) Capacity profile at 100 mA g^{-1} current density for 100 cycles; (b) Rate performance and (c) long-cycling for 2000 cycles at 2000 mA g^{-1} current density.

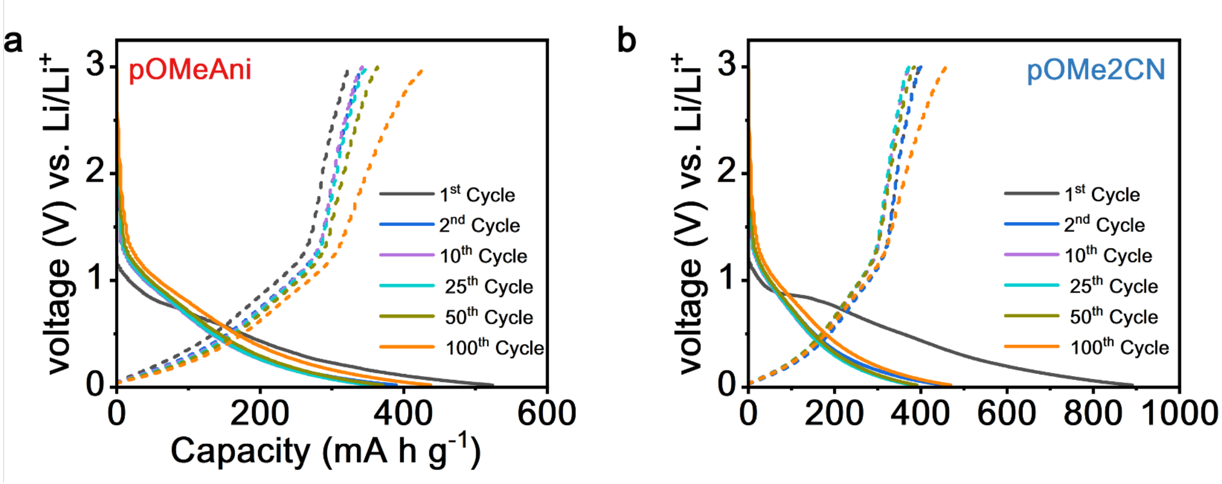

Figure S9. Galvano static charge-discharge curves at 100 mA g^{-1} current density of 20% anodes: (a) **pOMeAni** and (b) **pOMe2CN**.

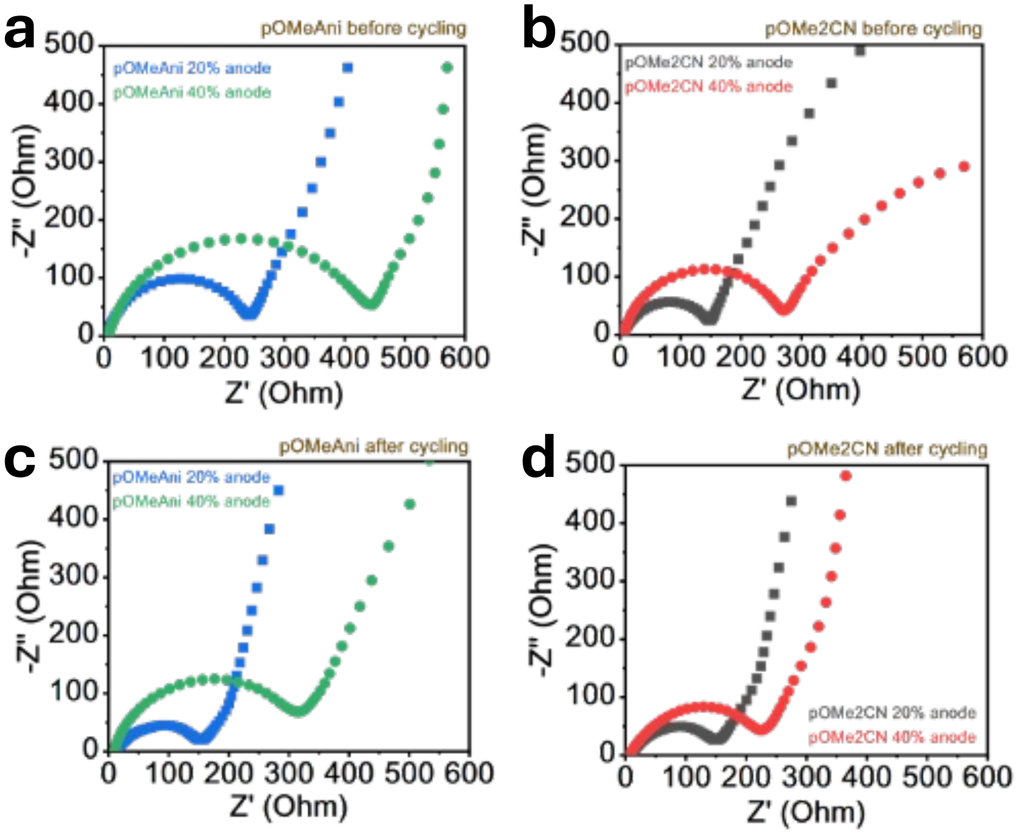

Figure S10. Nyquist plot of 20%/40% anodes at before 100 cycles: (a and c) **pOMeAni** and (b and d) **pOMe2CN**.

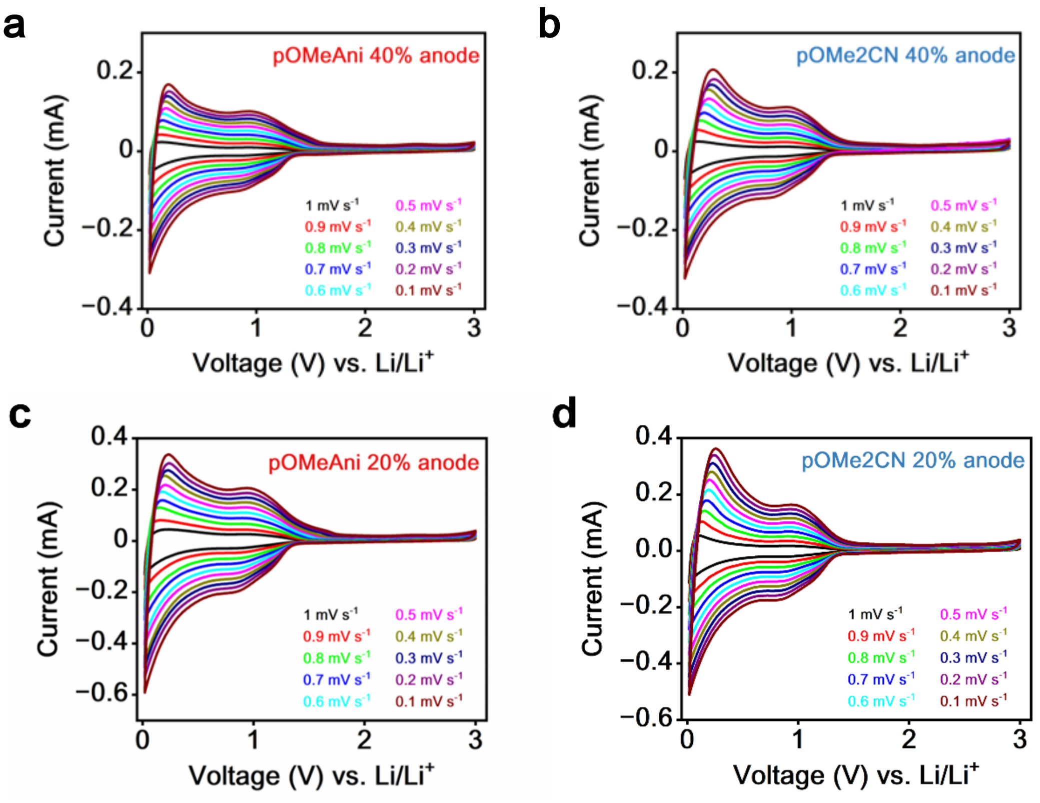

Figure S11. Sweep Rate CV: **pOMeAni** (a) 40%, (c) 20% and **pOMe2CN** (b) 40%, (d) 20%.

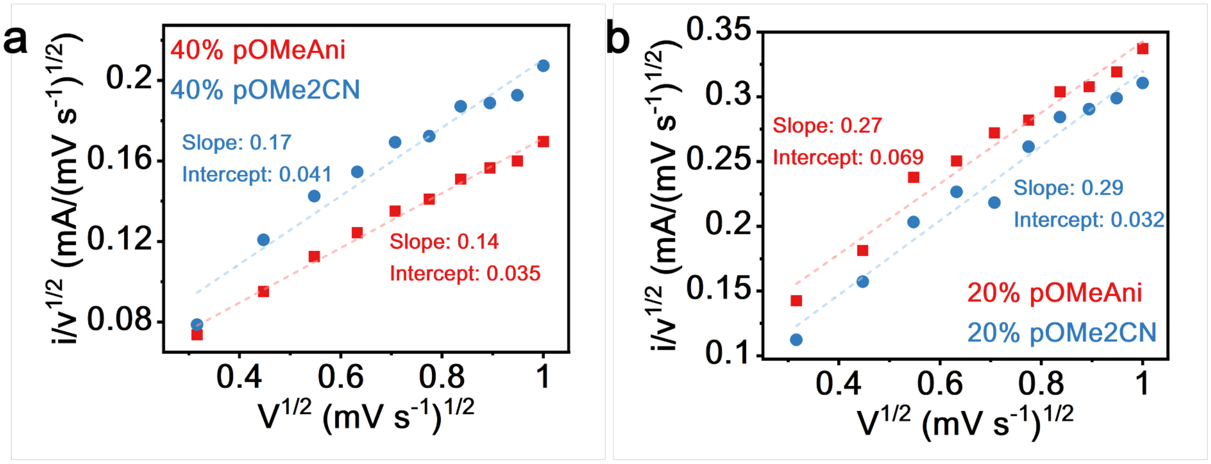

Figure S12. The $i/v^{1/2}$ vs $v^{1/2}$ plot. (a) 40% and (b) 20% anodes.

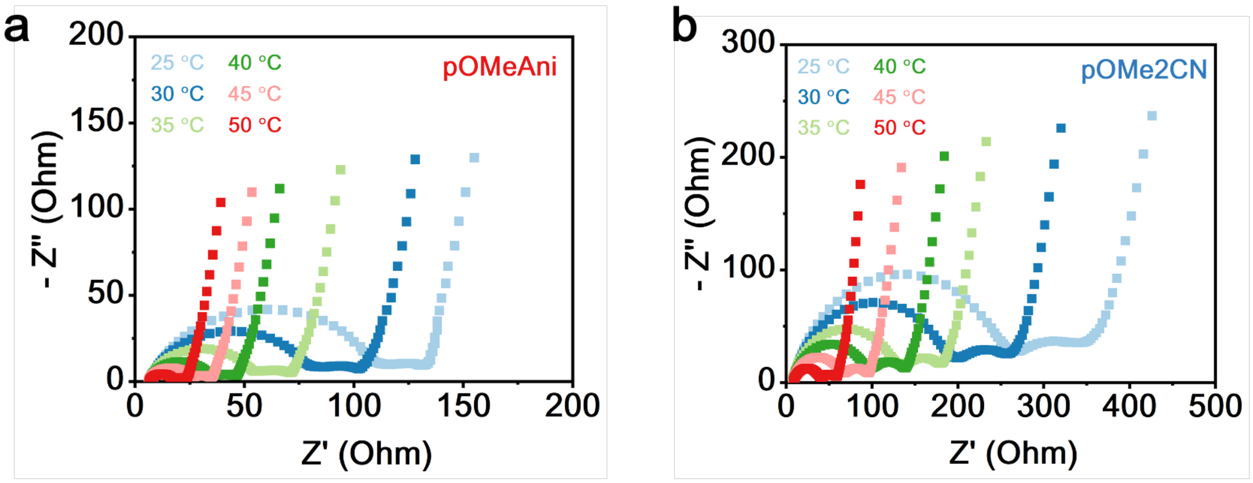

Figure S13. Nyquist plot of triazine-based anodes at various temperatures: (a) **pOMeAni** and (b) **pOMe2CN.**

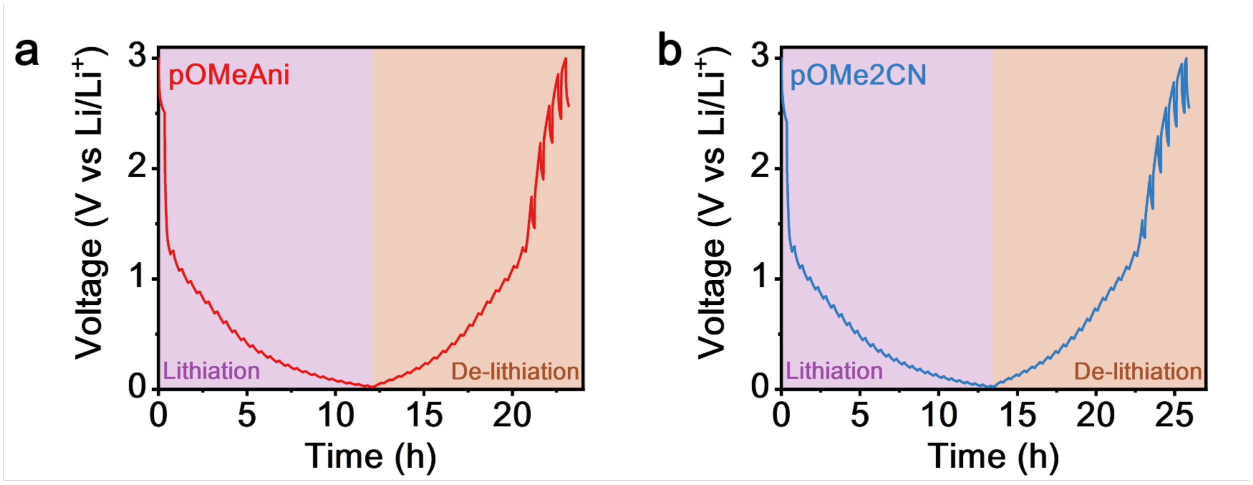

Figure S14. Galvanostatic intermittent titration technique (GITT) curves: (a) **pOMeAni** and (b) **pOMe2CN**.

Table S1. Summary of organic materials for LiB anodes.

Material Type	Compound Name	Voltage Window (V vs Li/Li$^+$)	Current density (mA g^{-1})	Specific Capacity (mA h g^{-1})	Ref.
Conjugated pores polymer	PDCzBT	0.0 - 3.0	100<br>200	404 (100 cycles)<br>312 (400 cycles)	1
	Polydiaminophenylsulfone-triazine	0.0 - 3.0	100<br>1000	565 (100 cycles)<br>375 (100 cycles)	2
	SNW-1/CNTs	0.01 - 3.0	500	203 (1000 cycles)	3
Polyimide	PI	0.001 - 3.0	100	578 (50 cycles)	4
	PMAQ	0.01 - 3.0	500	465 (150 cycles)	5
	N-CPIMs	0.0 - 3.0	10	500 (5 cycles)	6
Polythiophene	P(C-TDPP-TA)<br>P(F-TDPP-TA)<br>P(C-TDPP-H)<br>P(F-TDPP-H)	0.01 - 3.0	100	357 (500 cycles)<br>298 (500 cycles)<br>327 (500 cycles)<br>278 (500 cycles)	7
	PTp-COOH	0.01 - 3.0	500	147 (1000 cycles)	8
	P3-AQT	0.0 - 3.5	50<br>100	380 (100 cycles)<br>400 (100 cycles)	9
	Poly-triazine-Thiopene (PTT)-1<br>Poly-triazine-Thiopene (PTT)-2<br>Poly-triazine-Thiopene (PTT)-3<br>Poly-triazine-Thiopene (PTT)-4	0.01 - 3.0	100	495 (300 cycles)<br>671 (300 cycles)<br>707 (300 cycles)<br>772 (300 cycles)	10
	DBD-CMP-1<br>DBD-CMP-2	0.005 - 3.0	100	315 (300 cycles)<br>595 (300 cycles)	11

Polystyrene	Hyper-crosslinked polystyrene	0.0 - 3.0	200<br>2000	356 (100 cycles)<br>222 (1000 cycles)	12
Triazine dendrimer	DAP	0.02 – 3.0	500	444 (100 cycles)	13
Triazine micropores polymer	ACT	0.02 – 3.0	5000	247 (4000 cycles)	14
	rCTF	0.005-3	300<br>12000	1190 (500 cycles)<br>500 (1500 cycles)	15
	Si@NPC	0-1.5	500<br>16000	1390 (200 cycles)<br>420 (200 cycles)	16
	CNT@TAPT-BTPA	0.02-3	1000<br>100	350.9 (2000 cycles)<br>957.5 (50 cycles)	17
	TzThBT	0.005-3	500<br>5000	775 (500 cycles)<br>326 (1500 cycles)	18
Triazine small compound	**pOMeAni**	0.02 – 3.0	100<br>2000	437 (100 cycles)<br>432 (2000 cycles)	**This Work**
	**pOMe2CN**	0.02 – 3.0	100<br>2000	468 (100 cycles)<br>445 (2000 cycles)	

References

(1) Zhang, S.; Huang, W.; Hu, P.; Huang, C.; Shang, C.; Zhang, C.; Yang, R.; Cui, G. Conjugated microporous polymers with excellent electrochemical performance for lithium and sodium storage. *J. Mater. Chem. A* **2015**, *3* (5), 1896-1901.

(2) Ma, Q.; Zheng, J.; Kang, H.; Zhang, L.; Zhang, Q.; Li, H.; Wang, R.; Zhou, T.; Chen, Q.; Liu, A.; Li, H.; Zhang, C. Conjugated Porous Polydiaminophenylsulfone–Triazine Polymer—A High-Performance Anode for Li-Ion Batteries. *ACS Appl. Mater. Interfaces* **2021**, *13* (36), 43002-43010.

(3) Xu, S.-X.; Xu, W.; Kong, L.-J.; Zhang, Y.-H. Amorphous N-rich organic polymer/carbon nanotube composites as effective anode material for advanced lithium ion batteries. *SN Appl. Sci.* **2020**, *2*, 199.

(4) He, J.; Liao, Y.; Hu, Q.; Zeng, Z.; Yi, L.; Wang, Y.; Lu, H.; Pan, M. Investigation of polyimide as an anode material for lithium-ion battery and its thermal safety behavior. *Ionics* **2020**, *26* (7), 3343-3350.

(5) Ba, Z.; Wang, Z.; Zhou, Y.; Li, H.; Dong, J.; Zhang, Q.; Zhao, X. Electrochemical Properties of a Multicarbonyl Polyimide Superstructure as a Hierarchically Porous Organic Anode for Lithium-Ion Batteries. *ACS Appl. Energy Mater.* **2021**, *4* (11), 13161-13171.

(6) Han, X.; Han, P.; Yao, J.; Zhang, S.; Cao, X.; Xiong, J.; Zhang, J.; Cui, G. Nitrogen-doped carbonized polyimide microsphere as a novel anode material for high performance lithium ion capacitors. *Electrochim. Acta* **2016**, *196*, 603-610.

(7) Xu, Z.; Hou, S.; Zhu, Z.; Zhou, P.; Xue, L.; Lin, H.; Zhou, J.; Zhuo, S. Functional thiophene-diketopyrrolopyrrole-based polymer derivatives as organic anode materials for lithium-ion batteries. *Nanoscale* **2021**, *13* (4), 2673-2684.

(8) Numazawa, H.; Sato, K.; Imai, H.; Oaki, Y. Multistage redox reactions of conductive-polymer nanostructures with lithium ions: potential for high-performance organic anodes. *NPG Asia Mater.* **2018**, *10* (5), 397-405.

(9) Zhang, C.; Chen, S.; Zhou, G.; Hou, Q.; Luo, S.; Wang, Y.; Shi, G.; Zeng, R. 3-Anthraquinone substituted polythiophene as anode material for lithium ion battery. *J. Electroanalytical Chem.* **2021**, *895*, 115495.

(10) Xue, X.; Luo, J.; Kong, L.; Zhao, J.; Zhang, Y.; Du, H.; Chen, S.; Xie, Y. The synthesis of triazine–thiophene–thiophene conjugated porous polymers and their composites with carbon as anode materials in lithium-ion batteries. *RSC Adv.* **2021**, *11* (18), 10688-10698.

(11) Yang, T.; Zhang, C.; Ma, W.; Gao, X.; Yan, C.; Wang, F.; Jiang, J.-X. Thiophene-rich conjugated microporous polymers as anode materials for high performance lithium- and sodium-ion batteries. *Solid State Ion.* **2020**, *347*, 115247.

(12) Li, Z.; Zhong, W.; Cheng, A.; Li, Z.; Li, L.; Zhang, H. Novel hyper-crosslinked polymer anode for lithium-ion batteries with highly reversible capacity and long cycling stability. *Electrochim. Acta* **2018**, *281*, 162-169.

(13) Baskoro, F.; Chiang, P.-C.; Lu, Y.-C.; Patricio, J. N.; Arco, S. D.; Chen, H.-C.; Kuo, W.-S.; Lai, L.-L.; Yen, H.-J. Columnar liquid-crystalline triazine-based dendrimer with carbon nanotube filler for efficient organic lithium-ion batteries. *Electrochim. Acta* **2022**, *434*, 141306.

(14) Tong, Y.; Wang, J.; Sun, Z.; Huang, W. Extremely-Long-Lifespan and Ultrahigh-Rate Li-Ion Batteries Using Conjugated Porous Triazine Polymers. *ACS Appl. Mater. Interfaces* **2023**, *15* (11), 14274-14281.

(15) Buyukcakir, O.; Ryu, J.; Joo, S. H.; Kang, J.; Yuksel, R.; Lee, J.; Ruoff, R. S. Lithium accommodation in a redox-active covalent triazine framework for high areal capacity and fast-charging lithium-ion batteries. *Adv. Funct. Mater.* **2020**, *30*(36), 2003761.

(16) Zhu, J.; Chen, M.; Qu, H.; Xie, J.; Zhang, Y.; Liu, L.; Zhou, H. Silicon Anodes Protected by a Nitrogen-Doped Porous Carbon Shell for High-Performance Lithium-Ion Batteries. *Nanoscale* **2017**, *9*(25), 8871–8878.

(17) Lian, L.; Li, K.; Ren, L.; Han, D.; Lv, X.; Wang, H. G. Imine-linked triazine-based conjugated microporous polymers/carbon nanotube composites as organic anode materials for lithium-ion batteries. *Colloids Surf., A* **2023**, *657*, 130496.

(18) Ren, S. B.; Ma, W.; Zhang, C.; Chen, L.; Wang, K.; Li, R. R.; Jiang, J. X. Exploiting polythiophenyl-triazine-based conjugated microporous polymer with superior lithium-storage performance. *ChemSusChem* **2020**, *13*(9), 2295–2302.
